# Ultrastructure and cytochemistry of intrauterine embryonic and larval stages of *Ityogonimus lorum* (Digenea: Brachylaimidae) involving transitory development of ciliated miracidia

**DOI:** 10.1007/s00436-020-06629-z

**Published:** 2020-02-27

**Authors:** Zdzisław Świderski, David Bruce Conn, Jordi Miquel

**Affiliations:** 1 Witold Stefański Institute of Parasitology, Polish Academy of Sciences, 51/55 Twarda Street, 00-818 Warsaw, Poland; 2 Department of Biology and One Health Center, Berry College, Mount Berry, GA 30149 USA; 3 Department of Invertebrate Zoology, Museum of Comparative Zoology, Harvard University, 26 Oxford Street, Cambridge, MA 02138 USA; 4 Secció de Parasitologia, Departament de Biologia, Sanitat i Medi Ambient, Facultat de Farmàcia i Ciències de l’Alimentació, Universitat de Barcelona, Av. Joan XXIII, sn, 08028 Barcelona, Spain; 5 Institut de Recerca de la Biodiversitat (IRBio), Universitat de Barcelona, Av. Diagonal, 645, 08028 Barcelona, Spain

**Keywords:** *Ityogonimus lorum*, Brachylaimidae, Intrauterine embryogenesis, Miracidial morphogenesis, Ultrastructure, Cytochemistry

## Abstract

Results of the present study provide ultrastructural evidence that miracidial morphogenesis is fully completed within the intrauterine eggs while in the most posterior uterine regions of *Ityogonimus lorum*, a digenean parasite of an Iberian mole, *Talpa occidentalis* (Eulipotyphla, Talpidae). Using transmission electron microscopy (TEM), the ultrastructural characteristics of diverse cell types and their organelles of these developing embryos and fully formed miracidia within the eggshell were examined. The eggshell and embryonic envelopes are similar to those described previously by many authors for other digeneans. However, the developing miracidia are unique among previously described digeneans in possessing transitory cilia during larvigenesis, but completely lacking cilia in fully formed miracidium larvae. The evidence for completion of miracidial maturation in intrauterine eggs is based on the presence of the following structures: (1) transitional stage of ciliated differentiating miracidial epithelium; (2) apical and lateral glands, characteristic for digenean miracidia; and (3) fully developed germinative cells grouped together in the germinative sac localized in the posterior region of the miracidium. The protonephridial system with its characteristic flame cells and the nervous system with diverse types of neurons and nerve centers, which are characteristic for other digenean species reported until now, are absent from all these developmental stages of *I. lorum*. Based on these observations, we hypothesize that the life cycle of *I. lorum* is entirely terrestrial, involving passive transmission by ingestion of eggs containing unciliated miracidia to the first intermediate host.

## Introduction

Studying digenean eggs actually involves examining three distinct stages of the life cycle: the adult, the embryo, and the miracidium larva, which consequently involves examination of the three distinct processes of fertilization, embryogenesis, and larvigenesis (Conn 2000). Since most studies are conducted in situ within the parent worm, and the uterus, Mehlis’ gland, vitellaria, and other reproductive organs plan a critical role in egg development, the adult must be examined. The eggshell itself is mostly a product of the adult, including the primary shell matrix derived from vitellocyte secretions. Yet, some of the egg coverings are derived from embryonic structures, the inner and outer embryonic envelopes (Conn et al. 2018). Some digeneans are oviparous and do not complete embryogenesis or larvigenesis inside the adult, but rather in the external environment. However, many species are ovoviviparous, completing both embryogenesis and larvigenesis within the parent’s uterus, and thus being released into the environment ready to hatch and swim to seek the host immediately. The best known of these are the schistosomes, which were among the first to be studied due to their medical importance (Świderski 1994a). Because most such digeneans must swim freely through water to locate their snail host, they are equipped with well-developed cilia for locomotion, sensory capabilities integrated by larval neurons, and a protonephridial system with flame cells for osmoregulation in the hypotonic environment (Świderski et al. 2010, 2014; Świderski and Conn 2014). However, some digeneans do not hatch in the water but hatch only inside the first intermediate host snail after ingestion. This is particularly true of those that complete their entire life cycle in a terrestrial environment, using terrestrial snails as intermediate hosts. Most members of the family Brachylaimidae that have been studied with regard to their life cycle are known or suspected to have such host sequences (Ulmer 1951a, b; Mas-Coma et al. 1986; Mas-Coma and Montoliu 1986, 1987, 1995; Butcher and Grove 2001; Gracenea and González-Moreno 2002; González-Moreno and Gracenea 2006; Segade et al. 2011; Sirgel et al. 2012; Nakao et al. 2017, 2018; Waki et al. 2020). Like many members of the family Brachylaimidae, *Ityogonimus lorum* belongs to this latter category.

Unfortunately, brachylaimids, like most digeneans, have highly resistant eggshells that are technically very difficult to study ultrastructurally due to their small size and especially the difficulty in getting fixatives and embedding resins to infiltrate through the thick tanned eggshell matrix. Our previous success in surmounting these difficulties (see review by Conn et al. 2018) enabled us to obtain suitably infiltrated and embedded eggs. Thus, we engaged in this study to determine whether the miracidia of *I. lorum* conform to a pattern of functional morphology and ultrastructure particularly suited to an exclusively terrestrial life cycle that would be likely in the case of trematodes that successfully use fossorial moles as definitive hosts.

## Materials and methods

### Specimens

Live specimens of *Ityogonimus lorum* (Dujardin, 1845) were isolated from the intestine of a naturally infected Iberian mole (*Talpa occidentalis* Cabrera, 1907) trapped accidentally in June 2016 during a vole pest control campaign in Priesca (Asturias, Spain).

### Transmission electron microscopy

Recovered adult flukes were rinsed with a 0.9% NaCl solution and fixed in cold (4 °C) 2.5% glutaraldehyde in a 0.1 M sodium cacodylate buffer at pH 7.4 for 2 h, rinsed in 0.1 M sodium cacodylate buffer at pH 7.4, post-fixed in cold (4 °C) 1% osmium tetroxide with 0.9% potassium ferricyanide in the same buffer for 1 h, rinsed in Milli-Q water (Millipore Gradient A10), dehydrated in an ethanol series and propylene oxide, embedded in Spurr’s resin, and polymerized at 60 °C for 72 h. Ultrathin sections (60–90 nm thick) were obtained using a Reichert-Jung Ultracut E ultramicrotome. Sections were placed on 200-mesh copper and gold grids. Sections placed on copper grids were double-stained with uranyl acetate and lead citrate according to the Reynolds procedure (Reynolds 1963). Stained ultrathin sections were examined in a JEOL 1010 transmission electron microscope operated at an accelerating voltage of 80 kV, in the “Centres Científics i Tecnològics” of the University of Barcelona (CCiTUB).

### Cytochemistry

Sections placed on gold grids were treated according to the Thiéry test (Thiéry 1967) to reveal the ultrastructural localization of glycogen. Thus, sections were treated in periodic acid (PA), thiocarbohydrazide (TCH), and silver proteinate (SP) as follows: 30 min in 10% PA, rinsed in Milli-Q water, 24 h in TCH, rinsed in acetic solutions and Milli-Q water, 30 min in 1% SP in the dark, and rinsed in Milli-Q water. Ultrathin sections were also examined in a JEOL 1010 transmission electron microscope in the CCiTUB.

## Results

Ultrastructure of the advanced stages of embryogenesis and miracidial larvigenesis in the eggshell-enclosed, intrauterine eggs of *Ityogonimus lorum* was examined by means of transmission electron microscopy (TEM) and TEM cytochemistry.

**Fig. 1:**
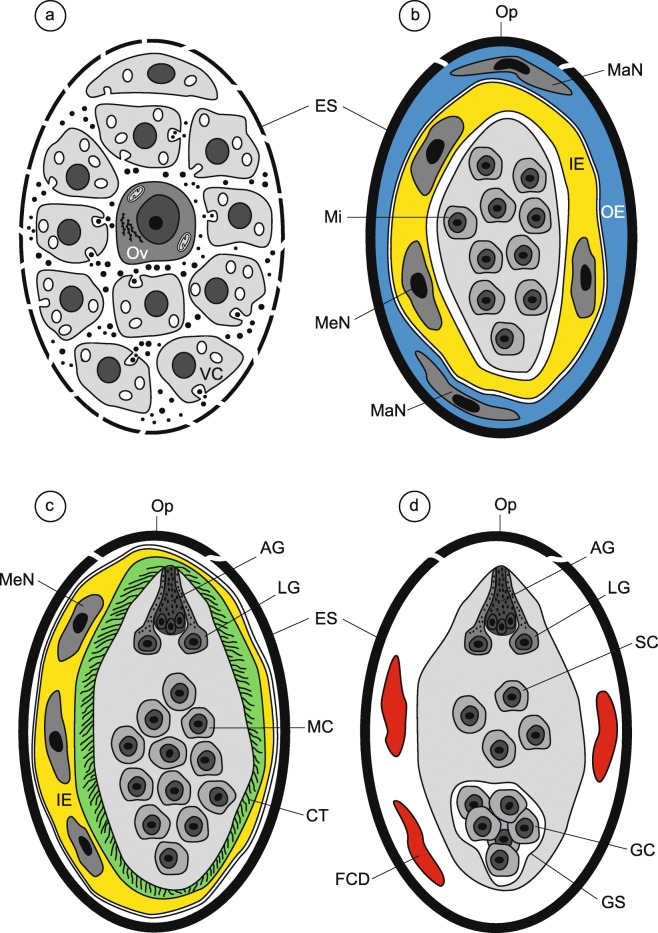
Four consecutive stages of embryogenesis and larvigenesis in the intrauterine eggs of *Ityogonimus lorum*. We added shading and coloration to aid in interpreting important features of each layer. **a** A newly formed egg of *I. lorum* in the ootype region, composed of a fertilized ovum (Ov) and several vitellocytes (VC), all surrounded by a thin discontinuous layer of the differentiating eggshell (ES). **b** The early embryo composed of several micromeres (Mi) in the stage of forming two embryonic envelopes, the outer (OE) and inner (IE) envelopes. **c** Advanced ciliated stage of miracidial differentiation and maturation. Note the presence of ciliated tegument (CT), a single apical gland (AG), two lateral glands (LG), and numerous undifferentiated miracidial cells (MC). **d** Final stage of mature non-ciliated larvae. Note the presence of numerous somatic miracidial cells (SC) and several germinative cells (GC), grouped together in a sac-like germinative follicle (GS). In this stage, note also the complete autolysis of the ciliated tegument of the miracidium, entirely eliminated in the presence of several lysosome-like structures, appearing as areas of focal degradation (FCD). MaN, macromere nucleus; MeN, mesomere nucleus; Op, operculum

**Fig. 2:**
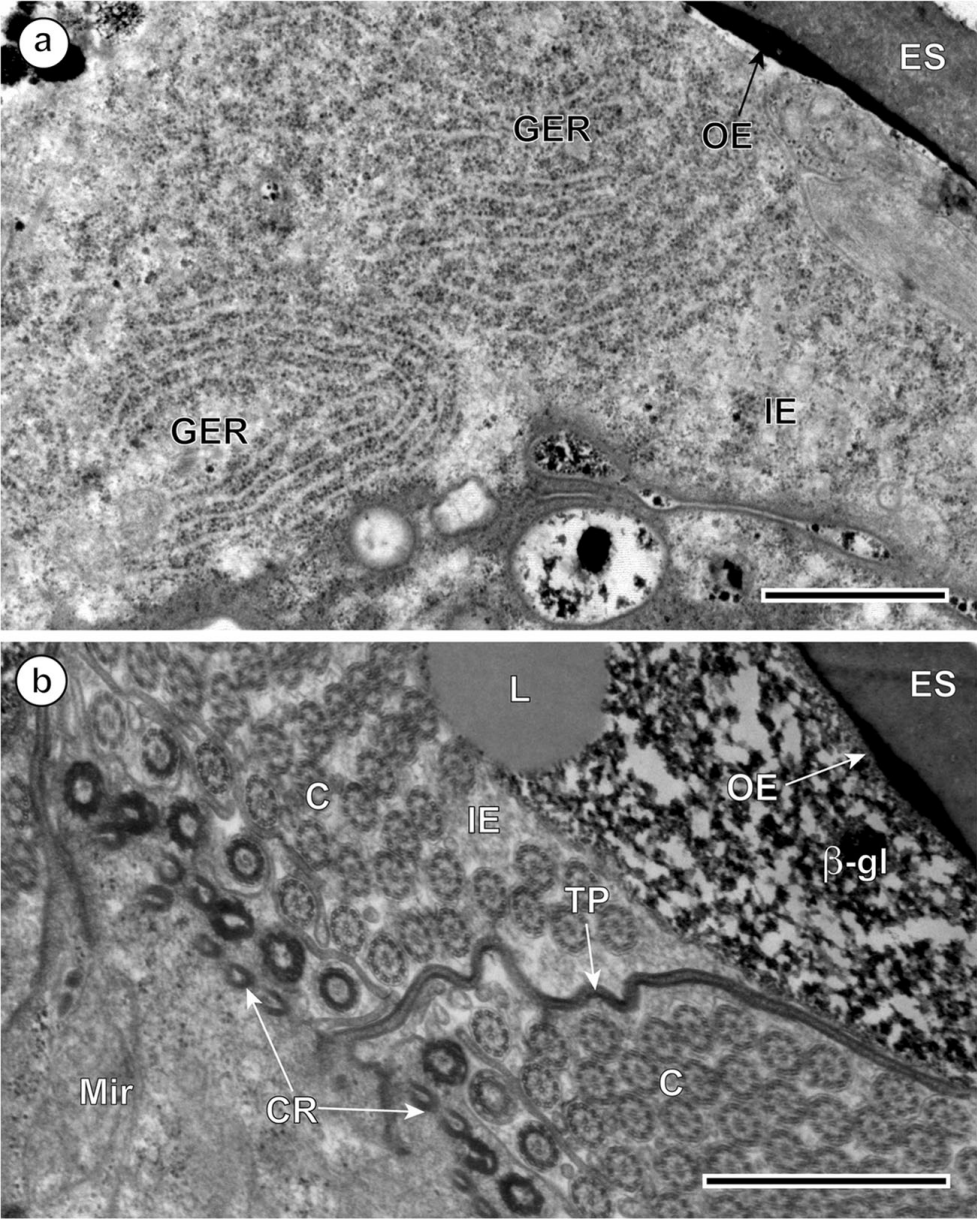
Ultrastructural details of *Ityogonimus lorum* egg envelopes. **a** Two large, concentric profiles of the granular endoplasmic reticulum (GER) in the cytoplasmic layer of the inner egg envelope (IE). The surface of embryonic tegument contains several vesicles of different sizes but is still unciliated. **b** A thick layer of the inner egg envelope (IE) cytoplasm with large, moderately saturated lipid droplets (L) and large accumulations of beta-glycogen particles (β-gl). The tegumental layer of the differentiating miracidium (Mir) shows presence of numerous ciliary rootlets (CR) and cilia (C), which are separated into individual zones by numerous tegumental processes (TP). ES, eggshell; OE, remnants of the outer envelope. Scale bars = 1 μm

**Fig. 3:**
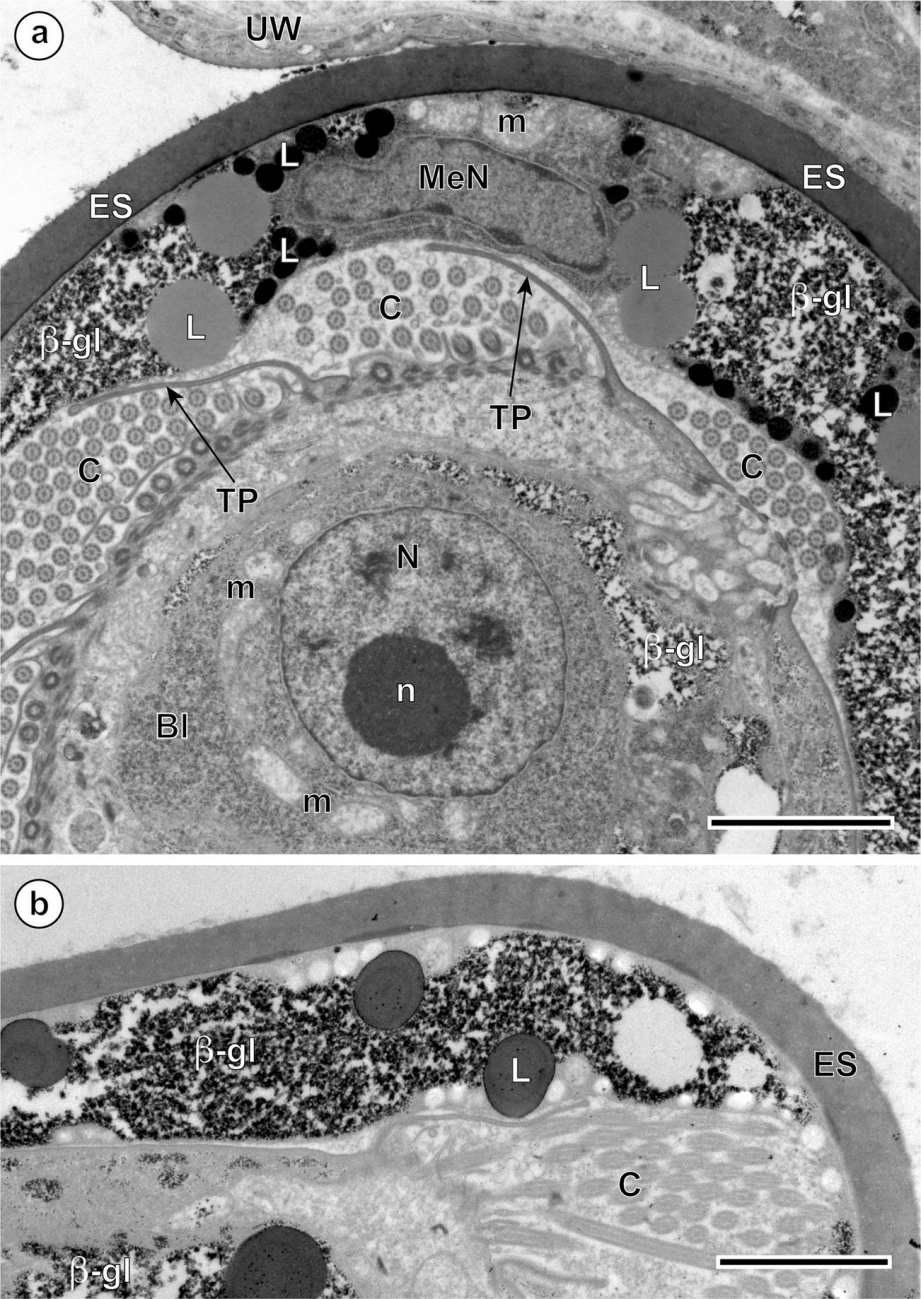
Intrauterine eggs of *Ityogonimus lorum* illustrating part of the advanced ciliated stage of the miracidium in the advanced stage of differentiation and maturation. **a** Egg with the larval surface illustrating the transitory development of cilia on the tegumental larval surface, with zones of cilia (C) which are separated by zones of thin, elongated tegumental processes (TP). **b** Specific cytochemical localization of beta-glycogen particles (β-gl) in the inner egg envelope and in the somatic peripheral musculature of differentiating larvae after application of the test of Thiéry. Bl, blastomere; ES, eggshell; L, lipid droplets; m, mitochondrion; MaN, macromere nucleus; MeN, mesomere nucleus; n, nucleolus; N, nucleus; UW, uterine wall. Scale bars = 2 μm

**Fig. 4:**
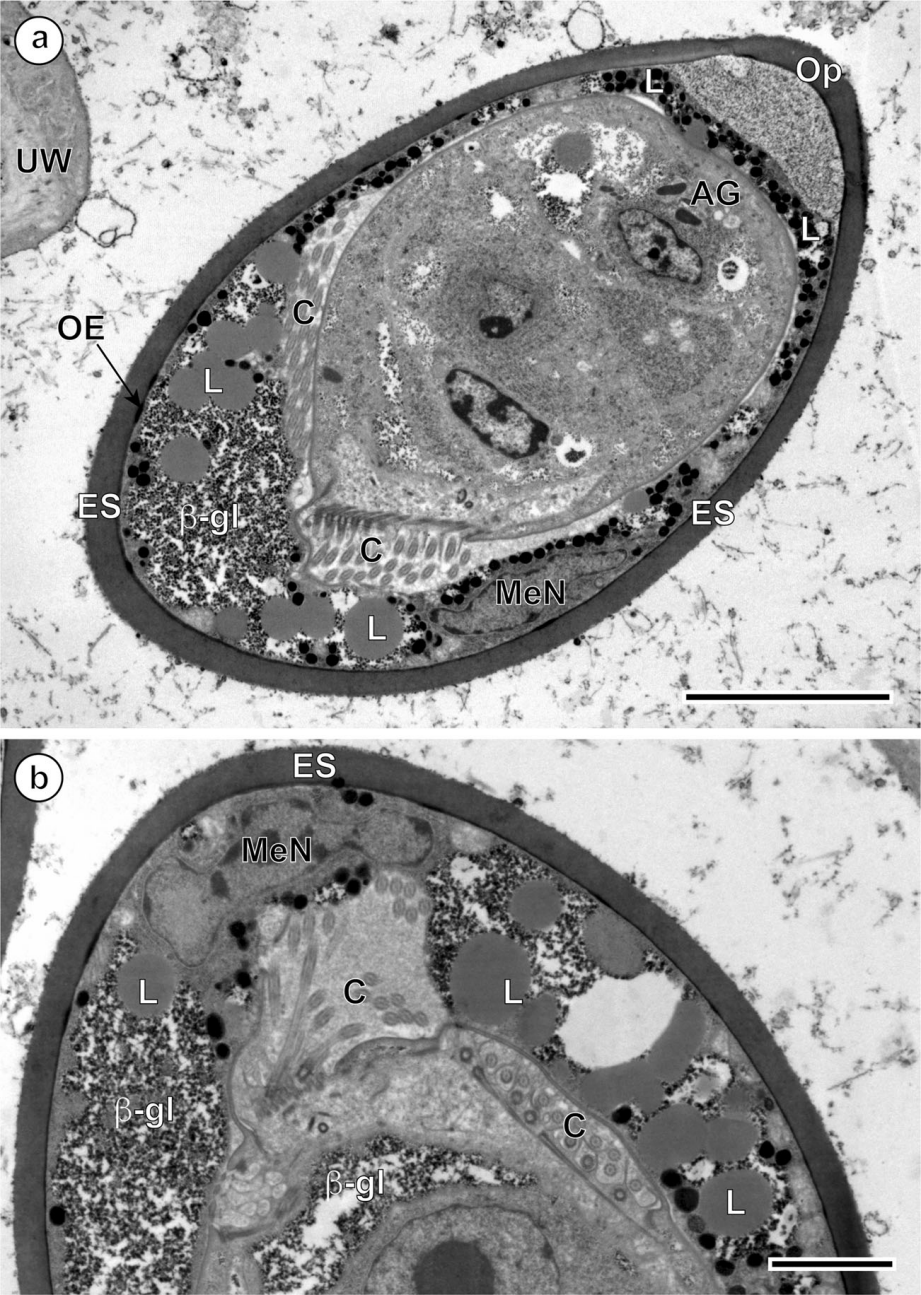
Ultrastructural details of a progressive degeneration of miracidial cilia at one pole of the *Ityogonimus lorum* eggs. **a** Presence of the unciliated, smooth miracidial surface of the operculum-oriented pole of the majority of fully differentiated larvae. **b** Similar observations at higher magnification, which shows presence of only very limited number of cilia (C) at the pole of egg situated opposite the operculum. AG, apical gland; β-gl, beta-glycogen particles; ES, eggshell; L, lipid droplets; MeN, mesomere nucleus; OE, outer envelope; Op, operculum; UW, uterine wall. Scale bars (**a**) = 5 μm, (**b**) = 2 μm

**Fig. 5:**
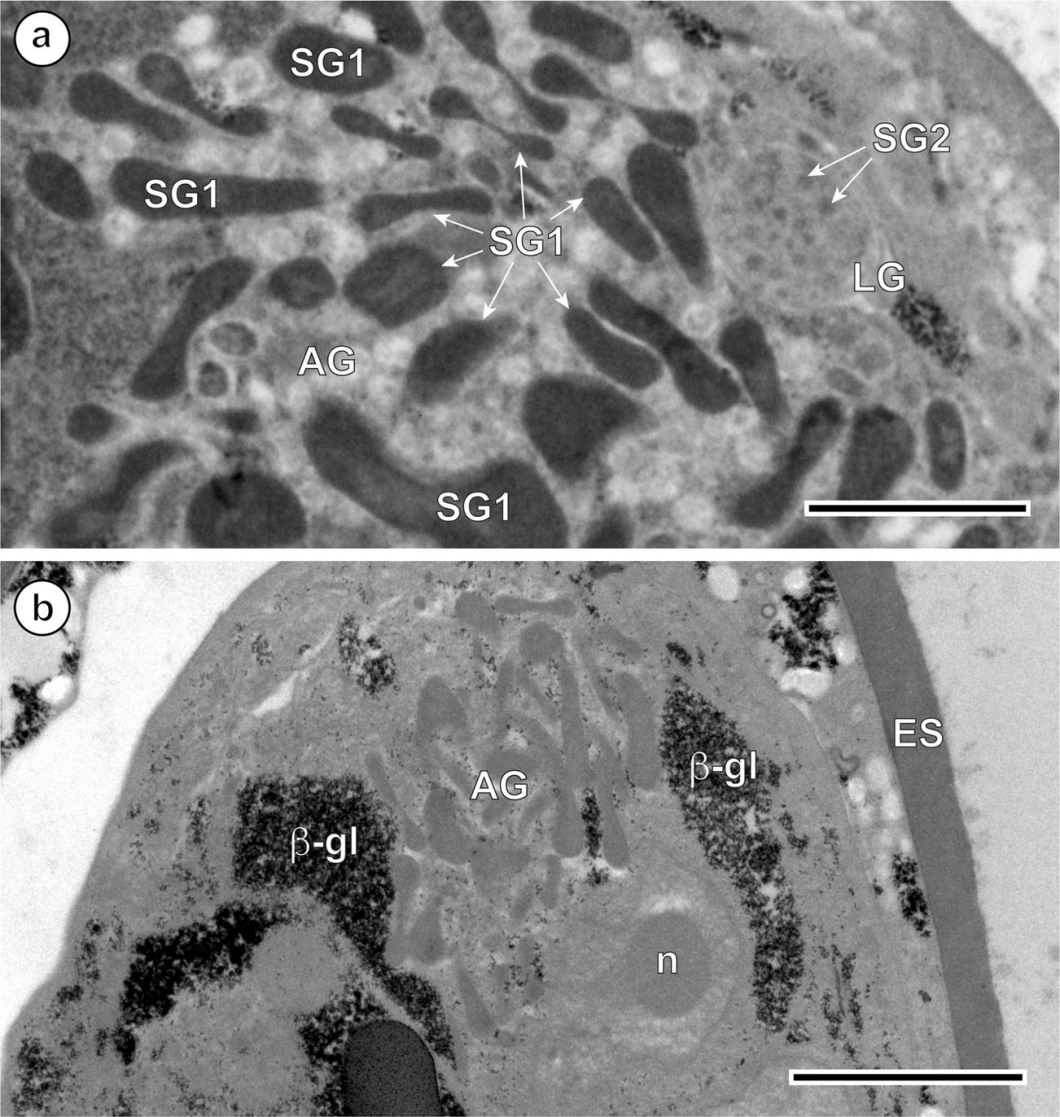
Ultrastructural details of the two types of miracidial glands. **a** Micrograph showing differences between large, elongated secretory granules SG1 of the apical gland (AG) and spherical, much smaller and evidently less electron-dense granules SG2 of the lateral gland (LG). **b** Details of the perinuclear region of the apical gland after the test of Thiéry. Note a large nucleus of the apical gland with a prominent, spherical nucleolus (n) surrounded by a thick layer of granular cytoplasm containing several dense accumulations of beta-glycogen particles (β-gl). ES, eggshell. Scale bars (**a**) = 1 μm, (**b**) = 2 μm

**Fig. 6:**
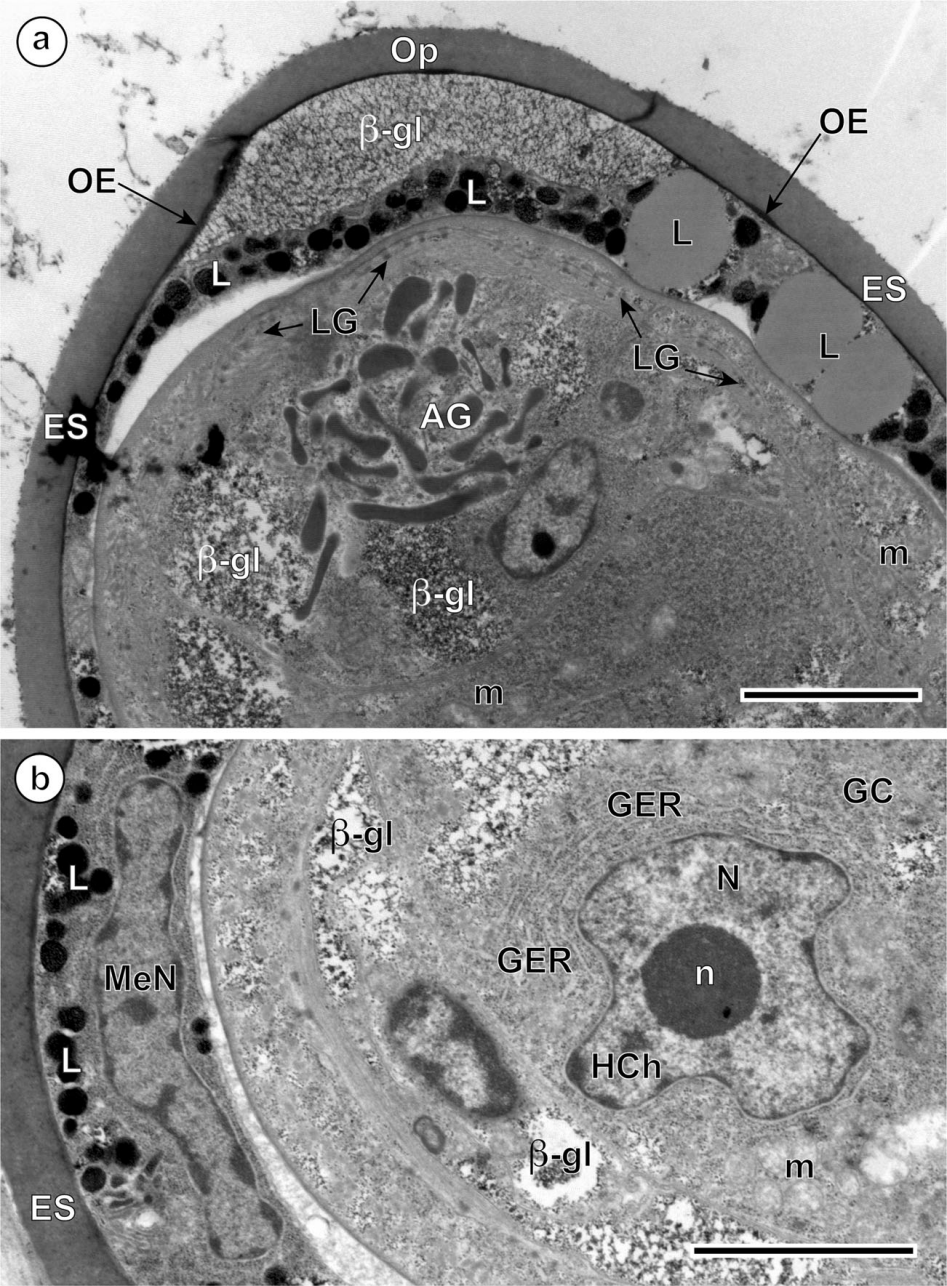
Ultrastructural details of mature unciliated, smooth-surfaced eggs of *Ityogonimus lorum*. **a** Apical gland (AG) with its characteristic, elongated secretory granules. **b** A germinative cell (GC) with large irregularly shaped nucleus (N) and a prominent, electron-dense nucleolus (n). β-gl, beta-glycogen particles; ES, eggshell; L, lipid droplets; GER, granular endoplasmic reticulum; LG, lateral glands; m, mitochondria; MeN, mesomere nucleus; OE, outer envelope; Op, operculum. Scale bars = 2 μm

The four diagrams on Fig. 1a–d illustrate and summarize the four consecutive stages of embryogenesis and larvigenesis observed in the intrauterine eggs of *I. lorum*. Figure 1a illustrates the ultrastructure of a newly formed egg of *I. lorum* in the ootype region, which is composed of a fertilized ovum and several vitellocytes all surrounded by a thin, malleable, and discontinuous layer of the differentiating eggshell. The proximal region of the uterus usually contains the eggs in the early stages of their embryonic development (Fig. 1b), composed of several micromeres, which will form the miracidium, as well as macro- and mesomeres participating in the formation of the outer and inner envelopes of the embryo. In this stage, the presence of three surrounding layers was observed: (1) a thick, electron-dense layer of the eggshell; (2) an outer envelope containing two large, slightly flattened nuclei of the macromeres, the cytoplasm of which is forming by fusion into a syncytium; and (3) the three, slightly smaller and less elongated nuclei of the mesomeres, the cytoplasmic fusion of which produces a thick layer of the inner embryonic envelope. In *I. lorum*, as in numerous other trematodes, the outer envelope with its two flattened nuclei of macromeres disappears rapidly and its place is taken by a rapidly growing inner envelope which accumulates great amounts of nutritive reserves in the form of large lipid droplets and dense agglomerations of beta-glycogen particles (Figs. 2b, 3a, b, 4a, b, 5b, and 6a). The remnants of the outer egg envelope are present and situated just under the eggshell (Fig. 2a, b). Large, concentric profiles of the granular endoplasmic reticulum in the cytoplasmic layer of the inner envelope are visible during an early premiracidial stage of embryogenesis (Fig. 2a). The surface of embryonic tegument contains several vesicles of different sizes, but is still unciliated (Fig. 2a).

In the advanced ciliated stage of the miracidium, a thick layer of the inner envelope cytoplasm with large, moderately saturated lipid droplets and large accumulations of beta-glycogen particles in the cytoplasm of this egg envelope is also observed (Figs. 2b and 3a, b). The tegumental layer of the differentiating miracidium (Fig. 1c) shows the presence of very numerous ciliary rootlets with cilia arising from them, which are separated into individual zones by numerous thin, very long tegumental processes (Figs. 2b and 3a, b). The outer envelope, usually situated between the eggshell and the inner envelope, is no longer visible in this stage (Fig. 3a), but a thick syncytial layer of the inner envelope with large, flattened nuclei of mesomeres, three primary blastomeres which formed initially this egg envelope, usually persists in this layer until advanced stages of embryogenesis (Fig. 3a, b). Numerous lipid droplets and large accumulations of beta-glycogen particles in the cytoplasm of this egg envelope are also observed (Figs. 2b and 3a, b).

**Fig. 7:**
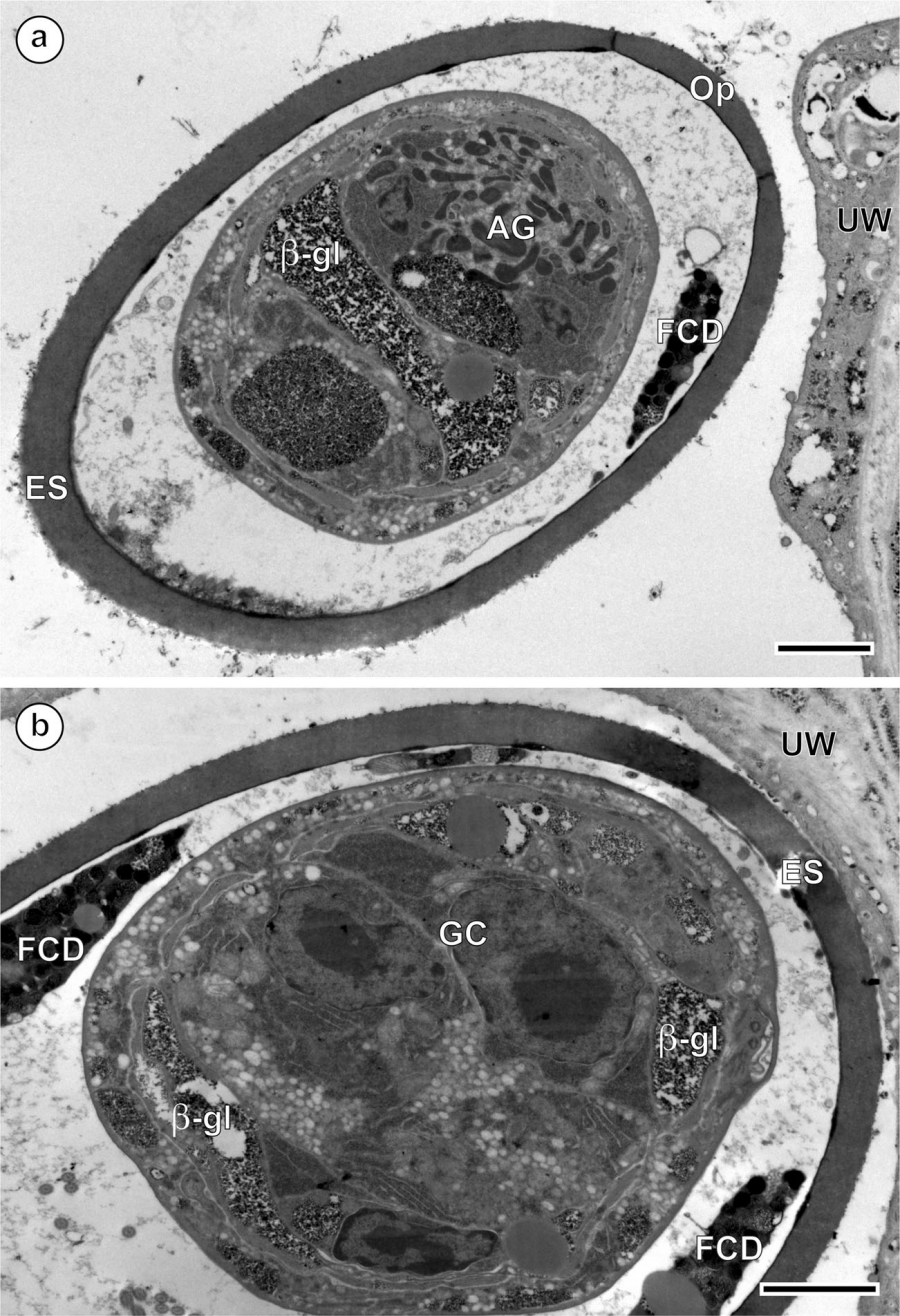
**a**, **b** Eggs of *Ityogonimus lorum* in the final stage of mature intrauterine unciliated miracidia. Note several focal cytoplasmic degradation (FCD) structures between the eggshell (ES) and unciliated miracidial tegument. AG, apical gland; GC, germinative cells; Op, operculum; UW, uterine wall. Scale bars = 2 μm

In the advanced stage of differentiation and maturation, a progressive degeneration of miracidial cilia, advancing from one to the opposite pole of the egg, was observed (Fig. 4a, b). This progressive degeneration of cilia on the surface of the miracidial tegument starts much earlier than the autolytic processes of the inner egg envelope, apparently due to a lysosomal activity of the areas of focal cytoplasmic degradation, which appear only much later in the space between the eggshell and the already completely unciliated miracidial tegument (Fig. 7a, b). In the mature eggs, the areas of focal cytoplasmic degradation were frequently observed and may be involved in the autolysis of some embryonic structures. Much longer persistence of the inner envelope is also confirmed and illustrated in Fig. 4a, b and Fig. 6a, b, which show a great variety of cell organelles and inclusions, namely numerous large lipid droplets and dense accumulations of beta-glycogen particles, which represent important reserves of nutritive material for miracidial morphogenesis.

Two types of miracidial glands are present in the miracidia of *I. lorum*: the apical gland and two lateral glands (Figs. 1d, 4a, 5a, b, 6a, and 7a). The secretory granules of the apical gland are large and elongated (Fig. 5a), while the secretory granules of the lateral glands are spherical, much smaller, and less electron-dense (Fig. 5a). The apical gland has a large nucleus with a prominent, spherical nucleolus surrounded by a thick layer of granular cytoplasm containing several dense accumulations of beta-glycogen particles (Fig. 5b). The flame cells and nerve cells were never observed during miracidial morphogenesis or in the mature miracidia of *I. lorum*.

In the mature unciliated, smooth-surface eggs of *I. lorum*, the germinative cells are grouped together in a sac-like germinative follicle and situated in the medioposterior part of the larva, the germatophore (Fig. 1d). The germinative cells (Figs. 6b and 7b) contain a large irregularly shaped nucleus with prominent, large nucleoli and numerous electron-dense heterochromatin islands arranged in the form of a network or chain-like pattern and distributed mainly in the karyoplasm adjacent to the nuclear membrane. The thin layer of granular cytoplasm is rich in free ribosomes and contains short profiles of granular endoplasmic reticulum and a few small mitochondria. Both nuclear and cytoplasmic features of these cells indicate their great developmental potential for further growth and multiplication in following stages of the life cycle.

## Discussion

These results provide the first data ever reported on cellular and subcellular ultrastructure of developing embryos and larvae of a brachylaimid digenean, despite the fact that this family is common worldwide and has been studied extensively by many researchers (e.g., Mas-Coma and Montoliu 1986, 1987, 1995; Butcher and Grove 2001; Segade et al. 2011; Nakao et al. 2017, 2018). As with other digenean taxa, research on eggs, embryos, and miracidium larvae are rare due to the small size, short longevity of these stages, and technical difficulty of preparing the highly resistant intact eggs for electron microscopy, though there is a wealth of information from light microscopy (Conn 2000).

In many basic respects, the structure and formation of *Ityogonimus lorum* eggs are similar to those of other digeneans (Świderski et al. 2013a; Conn et al. 2018), aspidogastreans (Świderski et al. 2011, 2012), as well as polylecithal cestodes (Świderski 1994b, c; Conn and Świderski 2008; Młocicki et al. 2010), including eggs of the monozoic cestodes such as gyrocotylideans or caryophyllideans (Bruňanská et al. 2012; Levron et al. 2016). Early cleavage leads to separation of blastomeres by size class, with mesomeres and macromeres forming the two embryonic envelopes, and micromeres forming the miracidium larva. The eggshell, as with all trematodes and polylecithal cestodes, is formed in cooperation with the vitellocytes, which are required for eggshell formation to occur (Conn and Etges 1983; Świderski 1994a; Świderski et al. 2010, 2013a). Two pleurogenid digenean species, *Brandesia turgida* and *Prosotocus confusus*, have been shown to have an extra “cocoon” layer surrounding each embryo outside the eggshell (Świderski et al. 2013b, 2015), but the functional significance of the “cocoon” is not known and to date has been described only from these two members of one family (Świderski and Conn 2014; Świderski et al. 2014; Conn et al. 2018).

The absence of tegumental cilia in the fully developed miracidium is a feature that is unique among digenean species that have been studied ultrastructurally to date. Our data show that the developing miracidia do have tegumental cilia, and that these are restricted to limited discrete bands so that the full tegument is never ciliated as in most digeneans. Even these small zones of cilia are transitory during larvigenesis, undergoing complete atrophy by the end or miracidial development inside the eggshell, and thus never functioning for locomotion. This has not been described previously for any species of digenean. However, it is similar to many cases of ontogenetic development of structures followed by subsequent atrophy of those structures during early development across the animal kingdom, including vertebrates as described in the classic treatise on evolution by Haeckel (1874).

It is noteworthy that our results show that in *I. lorum*, the miracidial cilia disappear earlier than the cellular atrophy of the inner egg envelopes, which persist much longer beyond completion of larvigenesis (see Fig. 6a, b). Also, apparently the miracidial cilia disappear progressively, with disappearance starting from one pole of the egg and continuing to the opposite pole on which they are still visible for much longer. Thus, while one pole of the developing miracidium still has cilia until the final stages of larvigenesis, the opposite pole is unciliated from early in larvigenesis (see Fig. 4a, b). For this reason, it is critical that investigators examine the full range of developmental stages when reporting on egg and miracidial structure.

The absence of a protonephridial system in *I. lorum* is unique among digeneans, and may reflect an exclusively terrestrial life cycle. The capacity for hypertonic osmoregulatory activity in a hypoosmotic freshwater environment is the norm among most digenean miracidia and cercariae, both of which are free-swimming in search of their host in most species. Thus, both of these digenean stages are generally equipped with flame cells and excretory ducts, in both freshwater and marine environments. Although the life cycle of *I. lorum* is still unknown, our proposal (see below) posits a life cycle in which this species is never outside a host, and thus can rely on the molluscan and mammalian hosts exclusively for their osmoregulatory needs.

Among cestodes, the primarily terrestrial cyclophyllideans typically lack ciliated motile oncosphere stages and also lack protonephridia in the hexacanth (Rybicka 1966; Jabbar et al. 2010), while other primarily aquatic groups such as pseudophyllideans and bothriocephalideans have both of these features (Świderski 1994b), which may be homologous to comparable structures in digeneans (Świderski 1994c). In the context of the present report, it is noteworthy that the strictly aquatic bothriocephalidean cestode, *Eubothrium salvelini*, has been shown to lack both a ciliated inner embryonic envelope (Świderski et al. 2005) and also protonephridia in the fully formed hexacanth (Młocicki et al. 2010). Thus, the absence of these features, and thus a typical bothriocephalidean coracidium stage, is not always associated with a terrestrial life cycle in the cestodes, but in the case of *E. salvelini* may be more related to the advanced ovoviviparous development of thin-shelled eggs in that species.

**Fig. 8:**
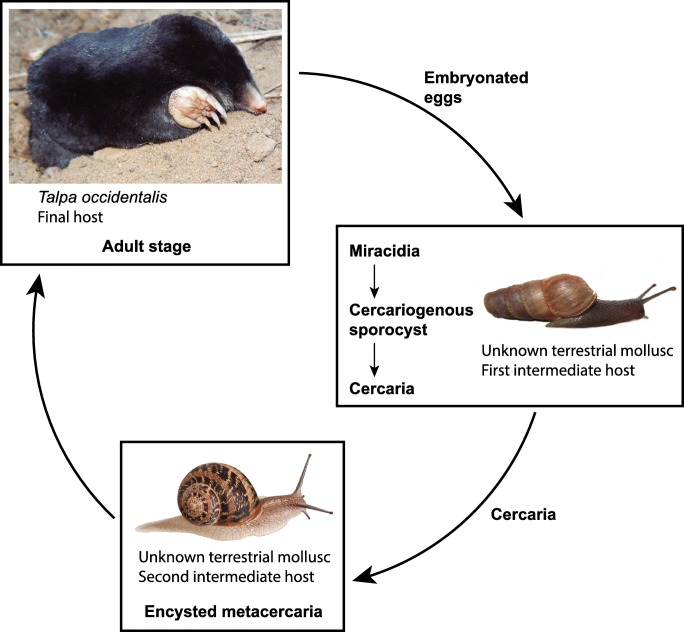
Diagram of hypothetical life cycle of *Ityogonimus lorum*, based on information available to date, including our data on functional ultrastructure of the miracidia

The miracidium of *I. lorum* is also distinctive among digenean species studied thus far in lacking any element of the nervous system. Similarly, apparent lack of neuronal elements has been described recently for the pleurogenid, *B. turgida* (Świderski et al. 2014). As with the lack of a protonephridial system, we propose that this is related to the hypothetical life cycle of *I. lorum* that avoids any active host-seeking behavior in the miracidium. In this regard, it is important to note that the eggs of both *B. turgida* and *I. lorum* are ingested by their snail host, and thus do not need neural integration of sensory input. However, while *B. turgida* infects freshwater snails, and thus may require protonephridia, we hypothesize that *I. lorum* infects terrestrial snails and thus may not require protonephridia (see Table 1 and Fig. 8).

**Table 1:** Comparison of the mode of infection and infective strategies of *Ityogonimus lorum* miracidia and *Brandesia turgida* miracidia

Digenean species:	
*Brandesia turgida* (Brandes, 1888) (Plagiorchioidea, Pleurogenidae)	*Ityogonimus lorum* (Dujardin, 1845) (Brachylaimoidea, Brachylaimidae)
Common developmental characteristics for both species: Their embryogenesis and entire miracidial larvigenesis takes place in the intrauterine eggs	
Definitive hosts:	
*Pelophylax esculentus* (Linnaeus, 1758), *Pelophylax lessonae* (Camerano, 1882), *Pelophylax ridibundus* (Pallas, 1771), *Rana temporaria* Linnaeus, 1758 (Amphibia, Anura, Ranidae)	*Talpa caeca* Savi, 1822, *Talpa europaea* Linnaeus, 1758, *Talpa occidentalis* Cabrera, 1907 (Mammalia, Eulipotyphla, Talpidae)
Intermediate hosts:	
Aquatic molluscs (*Bythinia*) act as first intermediate hosts and crustacean decapods or insects (Coleoptera, Odonata, Trichoptera) act as second intermediate hosts as in other pleurogenids with known life cycles?	Terrestrial molluscs (*Cernuella*, *Helix*, *Otala*, *Pseudotachea*, *Rumina*, *Theba*) act as first and second intermediate hosts as in all other brachylaimids with known life cycles?
Life cycle:	
Aquatic life cycle	Entirely terrestrial life cycle with three hosts as described in all other brachylaimids with known life cycles
Mode of infection:	
Passive, occurs by ingestion of eggs as in all other pleurogenids with known life cycles; characterized by the absence of neurons and nerve centers	Passive, occurs by ingestion of eggs as in all other brachylaimids with known life cycles
Infection strategies:	
- Absence of host-seeking behavior of miracidia - Ciliated miracidium - Protonephridial system: presence of two flame cells - Absence of neurons and nerve centers - The structural elements of egg envelopes and stored nutritive reserves are autolyzed and reabsorbed by the miracidia in the final stages of their larvigenesis	- Absence of host-seeking behavior of miracidia - Transitional presence of miracidial cilia and their atrophy in the mature miracidia - Protonephridial system: absence of flame cells - Absence of neurons and nerve centers - The structural elements of eggs envelopes and stored nutritive reserves are autolyzed and reabsorbed by the miracidia in the final stages of their larvigenesis

Based on the unique absence of three crucial host-seeking features in the fully formed miracidia of *I. lorum*, which lack tegumental cilia, protonephridia, and nervous system, we propose a life cycle as a hypothesis for future studies (Fig. 8). We propose here a cycle that would be completed entirely in a terrestrial environment and would involve two successive terrestrial gastropod hosts. The first would become infected by ingesting intact eggs containing the non-ciliated miracidia from the feces of an infected mole. In this mollusc first-intermediate host, the miracidium would undergo metamorphosis, developing into a sporocyst that would produce numerous cercariae. These would be shed into the mantle cavity of the snail and pass to the outside in the slime trail, from which the cercariae would be ingested by a second-intermediate host snail, in which they would encyst to form metacercariae. The next mole definitive host would become infected by eating the second-intermediate host snail.

Our hypothesis is further strengthened by extensive published research on other brachylaimid species. The first study was done by Ulmer (1951a, b) who reported extensive data on the life cycle of the related brachylaimid of rodents, *Postharmostomum helicis*, which has a similar two-gastropod life cycle. Ulmer’s *P. helicis* work in these two reports was reviewed and summarized with additional drawings of the life cycle and individual stages by Olsen (1974). Other authors have demonstrated similar two-gastropod cycles in more recent studies of brachylaimid life cycles, including among others *Brachylaima ruminae*, a parasite of European rodents (Mas-Coma and Montoliu 1986); *Dollfusinus frontalis*, a parasite of rodent and soricimorph mammals on Mediterranean islands (Mas-Coma and Montoliu 1987); *Pseudoleucochloridium pericardicum*, a parasite of soricimorphs in the French Pyrenees (Mas-Coma and Montoliu 1995); *Brachylaima aspersae*, a parasite of rodents in Spain (Segade et al. 2011); *Brachylaima ezohelicis*, a parasite of toads in Japan (Nakao et al. 2017); and *Brachylaima asakawai*, a parasite of rodents in Japan (Nakao et al. 2018).

All of these reports included drawings or light micrographs of eggs containing miracidia, apparently with reduced ciliation. However, all appeared to have some cilia, and thus are in contrast to our current report of *I. lorum*. It is important to stress that none of these reports employed transmission electron microscopy (TEM), so the identity of small structures such as cilia, which can be confirmed only ultrastructurally, was not possible. Furthermore, those life cycle studies lacking TEM did not examine whether protonephridia or neural cells were present. Thus, the present report is the only one to confirm unciliated miracidia and the first to report lack of protonephridia and neural cells. Future studies of egg ultrastructure in diverse brachylaimid species should address this.

The discovery of several unique features in the embryogeny and larval structure of *I. lorum* highlights how little we still know about the functional morphology of digeneans, as well as their developmental plasticity in response to diverse environments and strategies to access hosts. This provides a compelling argument for why more research is needed, especially across disparate taxa, and among the earliest stages of ontogeny. Goater et al. (2005) and Conn et al. (2008) presented a case for similar increase of study for the cercaria and post-cercaria stages of digeneans. Based on their interpretation of studies on the strigeid, *Ornithodiplostomum ptychocheilius*, they proposed recognition of three distinct developmental stages between the cercaria and adult of that species, and argued for more research and open reconsideration of what have been considered typical digenean life cycles (Conn 2007, 2010). Likewise, it is possible that the traditional thinking of miracidium function and role in the life cycle of digeneans has been overly simplified due to insufficient research on diverse taxa inhabiting diverse hosts in diverse environments.
